# Genetic differentiation of *Xylella fastidiosa* following the introduction into Taiwan

**DOI:** 10.1099/mgen.0.000727

**Published:** 2021-12-13

**Authors:** Andreina I. Castillo, Chi-Wei Tsai, Chiou-Chu Su, Ling-Wei Weng, Yu-Chen Lin, Shu-Ting Cho, Rodrigo P. P. Almeida, Chih-Horng Kuo

**Affiliations:** ^1^​ Department of Environmental Science, Policy and Management, University of California, Berkeley, CA 94720, USA; ^2^​ Department of Entomology, National Taiwan University, Taipei 106, Taiwan, ROC; ^3^​ Division of Pesticide Application, Taiwan Agricultural Chemicals and Toxic Substances Research Institute, Taichung 413, Taiwan, ROC; ^4^​ Institute of Plant and Microbial Biology, Academia Sinica, Taipei 115, Taiwan, ROC

**Keywords:** *Xylella fastidiosa*, genomic diversity, grapevines, Pierce’s disease, introduction event, Taiwan

## Abstract

The economically important plant pathogen *Xylella fastidiosa* has been reported in multiple regions of the globe during the last two decades, threatening a growing list of plants. Particularly, *X. fastidiosa* subspecies *fastidiosa* causes Pierce’s disease (PD) of grapevines, which is a problem in the USA, Spain, and Taiwan. In this work, we studied PD-causing subsp. *fastidiosa* populations and compared the genome sequences of 33 isolates found in Central Taiwan with 171 isolates from the USA and two from Spain. Phylogenetic relationships, haplotype networks, and genetic diversity analyses confirmed that subsp. *fastidiosa* was recently introduced into Taiwan from the Southeast USA (i.e. the PD-I lineage). Recent core-genome recombination events were detected among introduced subsp. *fastidiosa* isolates in Taiwan and contributed to the development of genetic diversity. The genetic diversity observed includes contributions through recombination from unknown donors, suggesting that higher genetic diversity exists in the region. Nevertheless, no recombination event was detected between *X. fastidiosa* subsp. *fastidiosa* and the endemic sister species *Xylella taiwanensis *, which is the causative agent of pear leaf scorch disease. In summary, this study improved our understanding of the genetic diversity of an important plant pathogenic bacterium after its invasion to a new region.

## Data Summary

All raw reads have been deposited in NCBI under BioProject PRJNA715299. Complete genome sequence of the reference isolate GV230 has been deposited in GenBank/ENA/DDBJ under the accession CP060159.

**Impact Statement**

The plant pathogen *Xylella fastidiosa* subspecies *fastidiosa* causes Pierce’s disease (PD) of grapevines and is a threat in multiple regions across North America, Europe, and Asia. In this work, we conducted population genomics analysis of this pathogen in Taiwan. Through sampling of districts with high disease prevalence and comparative analysis with isolates from elsewhere in the world, we confirmed that the Taiwanese populations were all originated from the Southeast USA. Following the invasion, mutation and recombination have contributed to the local expansion of genetic diversity. Intriguingly, although recombination between different subspecies of *X. fastidiosa* have been reported, we found no evidence of recombination between the Taiwanese *X. fastidiosa* and the endemic sister species *Xylella taiwanensis *. These findings provide important information regarding the global transmission of an important plant-pathogenic bacterium and improve our understanding of its evolution following invasion into a new region. Additionally, the newly reported genome sequences provide valuable resources for future studies of this pathogen.

## Introduction

Southeast Asia is a region of intricate tectonic and climatic evolution, complex biogeography, and a hotspot for global biodiversity [1–4]. Oceanic islands like Taiwan, are important contributors to plant richness within the region, with local diversity reflecting both palaeogeographic events and human-mediated plant movement [5]. Though there are many species endemic to Taiwan, approximately 73 % of vascular plants found in the island originated from neighbouring regions [6]. In recent decades, a significant number of plants from the Americas, Europe, and Africa have also been introduced. By the start of the new millennium, it is estimated that 341 plant species have been naturalized in Taiwan [7], many of them crops. In association with these novel crop species, numerous pests and pathogens have also been introduced [8, 9].

The economic losses caused by phytopathogen infections are estimated as $1 billion dollars worldwide every year [10]. In addition to the economic losses, emerging plant pathogens represent a threat to food security, not only by affecting a region’s capacity to meet its nutritional needs, but also by negatively impacting local economies and social structures dependent on trade [11–20]. The difficulties associated with the control and management of introduced plant diseases are well documented [21–23]. Specifically, the genetic homogeneity of crop monocultures favours the emergence of host-specialized pathogen genotypes and the increment of virulence [24, 25]. Furthermore, pathogens can quickly adapt to geographical and ecological conditions [26–29]. These problems are particularly concerning in the context of introduced plant pathogens with wide host ranges and demonstrated capacity to spill over to sympatric flora. In this regard, the xylem-limited bacterial plant pathogen *Xylella fastidiosa *, transmitted by various insect vectors, represents an important topic of study [30–33].

The species *X. fastidiosa* is taxonomically divided into three main monophyletic subspecies with allopatric origins: *X. fastidiosa* subsp. *fastidiosa *, native to Central America [34–36]; *X. fastidiosa* subsp. *multiplex *, native to temperate and subtropical North America [37, 38]; and *X. fastidiosa* subsp. *pauca *, thought to be native to South America [37]. Each of these subspecies has experienced a recent geographical expansion mediated by multiple intra- and inter-continental introduction events [30, 36, 39–42]. In the particular case of subsp. *fastidiosa*, that includes one introduction to the USA that led to the emergence of Pierce’s disease (PD) [36]. Within the USA, PD-causing isolates diversified into three phylogenetically supported clades. The clade PD-I is composed of isolates collected from the Southeast USA. The clades PD-II and PD-III are both composed of mostly Californian isolates, but each one includes isolates from a different location in the Southeast USA (i.e. Texas for PD-II and Georgia for PD-III) [36]. Subsequently, the introduction of PD-II-related isolates into the island of Mallorca in Spain has led to new disease outbreaks [39]. In addition, PD-causing isolates of subsp. *fastidiosa* have also invaded Taiwan [43].

The invasion of subsp. *fastidiosa* in Central Taiwan dates back to at least 2002, when the pathogen was first isolated from *Vitis vinifera* L. plants showing symptoms of PD [43]. Two genomic studies suggested that this invasion originated via the introduction of PD-I lineage from the Southeast USA [44, 45]. However, this hypothesis was based on analyses including only two isolates collected in Taiwan and requires further tests. The local studies of PD in Taiwan identified two xylem feeders (*Kolla paulula* and *Bothrogonia ferruginea*) as putative vectors [46, 47]. The local PD control strategies have mainly been centred around vector control, assessment of the habitats suitable for these vectors, eradication of infected plants and alternative host plants, and planting of healthy seedlings [48]. Notably, alternative hosts may function as genetic diversity hotspots of plant pathogens and contribute to the maintenance of vector populations, thus requiring attention in the control strategies [49].

There is another xylem-limited bacterium only found in Taiwan. Leaf scorch symptoms described in pear plants (*Pyrus pyrifolia cv*. Hengshan) in Central Taiwan were documented in the 1980s, and the appearance of disease symptoms was correlated with the presence of a xylem-limited fastidious bacterium [50]. Isolate PLS229 was obtained from symptomatic pear and sequencing of its 16S rRNA showed 99 % similarity with published *X. fastidiosa* isolates [51]. Subsequent analyses found that the average nucleotide identity (ANI) between PLS229 and *X. fastidiosa* representatives ranged between 83.4–83.9 %, suggesting that PLS229 represents a new species [52]. This species, named *Xylella taiwanensis *, is the only known sister taxon to *X. fastidiosa* within the same genus. Analysis of the complete genome of *X. taiwanensis* shows that this species shares only ~66–70 % of its gene content with *X. fastidiosa* [53].

Both *X. taiwanensis* and *X. fastidiosa* subsp. *fastidiosa* have a negative effect on Taiwanese crops. In the case of subsp. *fastidiosa*, vineyards located in hilly terrains are under high risk of PD infection. In an analysis of survey data from 2002 to 2012, infection rates of grapevines were highest in Waipu (71%), Tongxiao (73%), and Houli (85%) [48]. For comparison, in an adjacent district Zhuolan, only sporadic PD infections were reported and the infection rate was ~1 %. Regardless of the infection rates, the genetic diversity of subsp. *fastidiosa* isolates found in these districts and the degree of genotypic divergence among districts are unknown. Moreover, the prevalence of evolutionary events such as gene gain/loss, homologous recombination and mutation rate remain to be investigated. Addressing these points is important to understand how subsp. *fastidiosa* diversifies following an introduction event, and can aid the design and implementation of disease management strategies.

In this study, we conducted an exploratory sampling of the subsp. *fastidiosa* isolates across locations reported to have high PD incidence in Central Taiwan. Specifically, we aim to: (1) provide a first look of the genomic diversity of this pathogen in this region and infer the patterns of diversification, (2) confirm the source population of these Taiwanese isolates, (3) compare the genetic diversity and diversification patterns of the Taiwanese population with those sampled from elsewhere in the world, and (4) infer the levels of recombination among the Taiwanese subsp. *fastidiosa* isolates as well as with the endemic sister species *X. taiwanensis *.

## Methods

### Biological samples and genome sequencing

**Fig. 1.**
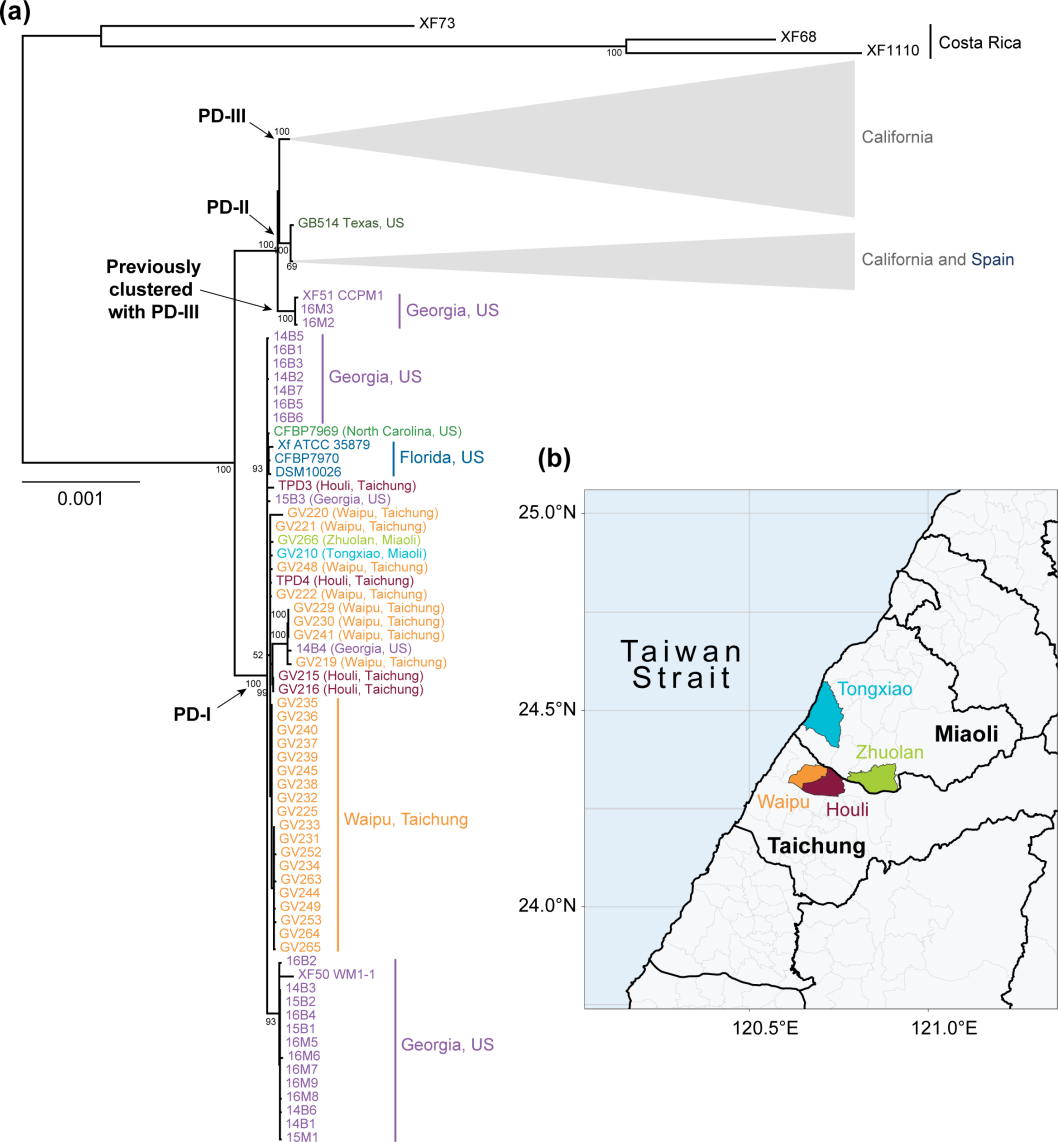
Relationship of PD-causing isolates. (a) Maximum-likelihood (ML) tree of worldwide PD-causing subsp. *fastidiosa* isolates. The phylogenetic inference was based on the core genome (i.e. shared by >99 % of the isolates) without removal of recombinant segments. Costa Rica isolates were used to root the tree. Clades encompassing California and Spain isolates have been compressed to their most recent common ancestor. The points of divergence for the PD-I to PD-III clades are indicated by arrows. Distinct colours are used to differentiate isolates from different geographic regions. (b) Collection locations of the Taiwan isolates.

Thirty-one field isolates were collected from symptomatic grapevines in Central Taiwan (Table 1, Fig. S1, available in the online version of this article). Most of these isolates were from Waipu (*n*=27), while few were from Houli (*n*=2), Tongxiao (*n*=1), and Zhuolan (*n*=1 Fig. 1b, Table 1). The bias reflects PD prevalence in these regions during our collection trips between 2015 and 2019. Notably, while PD infection rates were reported to be >70 % in Houli and Tongxiao during 2002–2012 [48], we had difficulties in finding infected grapevines in these two districts, suggesting that the local PD control strategies have been effective in recent years. After the infected plant samples were collected, the isolates were cultivated as described [43]. In short, petioles were surface-sterilized with 1 % bleach, rinsed with sterile water and then minced in PD2 broth to extract bacterial cells from xylem. The extract was streaked onto PD2 agar medium and incubated at 28 °C for 2–3 weeks in preparation for DNA extraction.

**Table 1.** Summary of assembly statistics for the newly sequenced Taiwanese *Xylella fastidiosa* subsp. *fastidiosa* isolates

Isolate	Geographic origin	Grapevine variety	Collection date	N50 (bp)	Genome size (bp)	Coverage (x)
GV210	Tongxiao, Miaoli	Kyoho	2015.08.21	87 160	2 463 940	294
GV215	Houli, Taichung	Kyoho	2017.06.30	99 605	2 463 006	331
GV216	Houli, Taichung	Kyoho	2017.08.03	97 514	2 468 540	285
GV219	Waipu, Taichung	Kyoho	2017.07.24	92 425	2 460 764	237
GV220	Waipu, Taichung	Kyoho	2017.07.24	99 605	2 461 512	288
GV221	Waipu, Taichung	Kyoho	2017.07.24	94 057	2 461 485	258
GV222	Waipu, Taichung	Kyoho	2017.07.24	113 299	2 461 579	254
GV225	Waipu, Taichung	Kyoho	2017.07.24	92 981	2 470 584	272
GV229	Waipu, Taichung	Golden Muscat	2017.07.24	99 551	2 465 605	232
GV230*	Waipu, Taichung	Black Queen	2018.06.24	2 514 993	2 514 993	277
GV231	Waipu, Taichung	Golden Muscat	2018.06.24	101 467	2 490 832	274
GV232	Waipu, Taichung	Golden Muscat	2018.06.24	90 280	2 467 990	272
GV233	Waipu, Taichung	Golden Muscat	2018.06.24	100 549	2 461 948	209
GV234	Waipu, Taichung	Golden Muscat	2018.06.24	100 555	2 463 675	213
GV235	Waipu, Taichung	Golden Muscat	2018.06.24	100 555	2 466 447	197
GV236	Waipu, Taichung	Golden Muscat	2018.06.24	100 555	2 465 286	215
GV237	Waipu, Taichung	Golden Muscat	2018.06.24	92 561	2 468 425	207
GV238	Waipu, Taichung	Golden Muscat	2018.06.24	90 280	2 516 844	285
GV239	Waipu, Taichung	Golden Muscat	2018.06.24	99 551	2 490 161	250
GV240	Waipu, Taichung	Golden Muscat	2018.06.24	124 734	2 469 727	237
GV241	Waipu, Taichung	Golden Muscat	2018.06.24	90 280	2 467 728	219
GV244	Waipu, Taichung	Golden Muscat	2018.12.25	80 059	2 774 440	57
GV245	Waipu, Taichung	Golden Muscat	2018.12.25	92 729	2 600 059	83
GV248	Waipu, Taichung	Black Queen	2019.07.03	98 380	2 456 198	19
GV249	Waipu, Taichung	Black Queen	2019.07.03	86 393	2 456 603	15
GV252	Waipu, Taichung	Golden Muscat	2019.07.03	90 026	2 455 757	16
GV253	Waipu, Taichung	Golden Muscat	2019.07.03	99 557	2 458 547	15
GV263	Waipu, Taichung	Black Queen	2019.12.04	97 977	2 460 836	14
GV264	Waipu, Taichung	Black Queen	2019.12.04	95 089	2 461 237	17
GV265	Waipu, Taichung	Black Queen	2019.12.04	99 087	2 457 696	16
GV266	Zhuolan, Miaoli	Kyoho	2019.12.31	86 635	2 459 085	16

*The reference isolate GV230 whose complete genome sequence is available.

The procedures for whole-genome shotgun sequencing and data processing were based on those described in our previous studies [45, 53]. All bioinformatics tools were used with the default settings unless stated otherwise. Briefly, the DNA samples of all 31 isolates were prepared using the Wizard Genomic DNA Purification Kit (A1120; Promega, USA). The Illumina sequencing libraries were prepared by the Genomic Technology Core (Institute of Plant and Microbial Biology, Academia Sinica) using KAPA LTP Library Preparation Kit (KK8232; Roche, Switzerland) and KAPA Dual-Indexed Adapter Kit (KK8722; Roche, Switzerland) with a target insert size of ~550 bp. The Illumina MiSeq paired-end sequencing service was provided by the Genomics Core (Institute of Molecular Biology, Academia Sinica) using the MiSeq Reagent Nano Kit v2 (MS-103–1003; Illumina, USA). For the reference isolate GV230, additional sequencing was conducted using the Oxford Nanopore Technologies (ONT) MinION platform (FLO-MIN106; R9.4 chemistry and MinKNOW Core v3.6.0) (Oxford Nanopore Technologies, UK). The sequencing library was prepared using the ONT Ligation Kit (SQK-LSK109) without shearing or size selection. Guppy v3.4.5 was used for base calling.

To obtain the complete genome sequence of the reference isolate GV230, the Illumina and ONT reads were combined for *de novo* assembly by using Unicycler v0.4.8-beta [54]. For validation, the Illumina and ONT raw reads were mapped to the assembly using BWA v0.7.12 [55] and Minimap2 v2.15 [56], respectively. The mapping results were programmatically checked using SAMtools v1.2 [57] and manually inspected using IGV v2.3.57 [58]. Gene prediction and annotation were performed using the NCBI prokaryotic genome annotation pipeline [59].

For the remaining 30 isolates, draft genome assemblies were prepared using only Illumina reads. The quality of raw paired FASTQ reads was evaluated using FastQC [60]. Low-quality reads and adapter sequences were removed from all paired raw reads using seqtk v1.2 (https://github.com/lh3/seqtk) and cutadapt v1.14 [61], respectively. After pre-processing, SPAdes v3.13 [62, 63] was used for *de novo* assembly with the -*careful* parameter and a -*k* of 21, 33, 55, and 77. Contigs were reordered with Mauve’s contig mover function [64] using the complete assembly of GV230 as a reference. Assembled and reordered genomes were then individually annotated using the Prokka pipeline [65].

### Additional genome sequences for comparative analysis

In addition to the 31 newly sequenced Taiwanese isolates, 175 genome sequences available from GenBank were included for comparative analysis (Table S1). These include two additional Taiwanese isolates from Houli (i.e. TPD3 and TPD4) [44], 171 isolates from the USA [45], and two isolates from Spain [45]. Those 171 USA isolates include 140 from California and 31 from Southeast USA, representing all three phylogenetic clades (i.e. PD-I, PD-II, and PD-III) established previously [45]. The sequence types (STs) of all isolates were determined based on an established multilocus sequence typing (MLST) scheme [66, 67] using BIGSdb [68].

Other than the combined data set of 206 *X *. *fastidiosa* subsp. *fastidiosa* isolates obtained from infected grapevines with PD symptoms, three Costa Rican subsp. *fastidiosa* isolates from non-*Vitis* hosts were included as the outgroup [34]. These three isolates are XF68 from *Psidium* sp., XF73 from *Coffea* sp., and XF1110 from *Vinca* sp. Furthermore, the type strain of *X. taiwanensis* (PLS229) [51–53] was included for between-species comparisons.

### Core genome alignments, construction of ML trees and recombination detection

Roary v3.11.2 [69] was used to identify the core, soft-core and accessory genomes among the isolates compared. The core genome was defined as genes shared by >99 % of the isolates, the soft-core was defined as genes shared by 95–99 % of the isolates, and the accessory genome was defined as genes shared by <95 % of the isolates. The analysis was performed separately for three sets of isolates, including: (1) a worldwide set containing all 206 PD-causing isolates (Table S1), (2) a Taiwan-only set containing 33 isolates. and (3) a PD-I/Taiwan set containing all 33 Taiwanese isolates and 28 PD-I isolates from Southeast USA (i.e. the putative source of the population in Taiwan). The ancestor/descendant relationships among populations were established using the worldwide PD-causing subsp. *fastidiosa* maximum-likelihood (ML) tree (see Results) as well as based on previous studies [44, 45]. For each set, genes in the core genome were aligned using Roary to produce a core-genome alignment. The alignment was used for ML phylogeny inference using RAxML [70]. The GTRCAT substitution model was used on tree construction, while tree topology and branch support were assessed with 1000 bootstrap replicates.

The core-genome alignment for the worldwide set was also used to estimate the location of recombinant events using fastGEAR [71]. Lineage-specific recombinant segments (ancestral) and isolate-specific recombinant segments (recent) were identified. Recombinant regions were removed from the alignment to generate a non-recombinant core genome of the worldwide set. This was done to determine the frequency of recombination events, to measure their impact on genetic diversity and the estimated mutation rate, and to estimate the contribution of recombination to genetic differentiation. After the recombinant regions were removed, this non-recombinant core was realigned using mafft [72] and used to construct a ML non-recombinant tree of the worldwide set as described. It should be noted that fastGEAR was designed to test recombination in individual gene alignments instead of core-genome alignments; yet, a previous study found that fastGEAR was more conservative than other recombination detection methods such as ClonalFrameML [36]. The number and location of recombinant regions was also estimated for the Taiwan-only and the PD-I/Taiwan sets. In both instances, recombinant segments were also removed. The location and origin of recombinant segments in the Taiwan-only set were visualized using fastGEAR. In addition, the presence of homologous recombination between the Taiwan-only set and *X. taiwanensis* was evaluated following the same protocol.

### Population genetic analyses, haplotype network assessment and population structure

Global measures of genetic diversity were estimated using the R package ‘PopGenome’ [73]. Briefly, PopGenome converts alignments into a biallelic matrix with rows corresponding to sequences and columns corresponding to single nucleotide polymorphism (SNP) positions. All SNPs were classified as 0 (major alleles) or 1 (minor alleles), while missing data and unknown variants were labelled as NA. The number of SNPs, nucleotide diversity (π), Tajima’s D [74], and Watterson’s estimator (*θ*) [75] were computed. Nucleotide diversity (π) measures the average number of nucleotide differences per site in pairwise comparisons among DNA sequences. Tajima’s D measures the frequency of polymorphisms present in a population and compares that value to the expectation under neutrality. The Watterson *θ* estimator measures the mutation rate of a population. The analyses were performed using core-genome alignments for the worldwide and PD-I/Taiwan sets, and then repeated using their corresponding non-recombinant cores. For each set, isolates were divided based on their geographic origins.

Haplotype networks were built for: (1) the non-recombinant PD-I/Taiwan set, (2) the recombinant Taiwan-only set, and (3) the non-recombinant Taiwan-only set. Core-genome haplotypes were calculated based on the number of mutations among the analysed isolates. The haplotype network was built using the ‘HaploNet’ function in the R package ‘pegas’ [76]. Haplotypes shapes and colours were coded by location. In addition, *SNP-sites* [77] was used to build a SNP alignment from the non-recombinant core-genome alignment for worldwide PD-causing isolates. This alignment was then used to evaluate if panmictic subpopulation clusters existed within the larger context of worldwide PD-causing isolates and to establish which isolates belonged to these potential subpopulations. The existence of genetically distinct subpopulations was assessed using a Bayesian analysis of population structure (BAPS) as implemented in the R package ‘hierBAPS’ [78]. This tool identifies population structure in haploid genomes by integrating over the allele frequencies, then uses this information to partition individual sequences into subpopulation clusters, followed by using the ‘ggtree’ package to annotate these clusters on a phylogenetic tree.

## Results

### The PD outbreak in Taiwan is linked to at least one introduction from the USA

**Fig. 2.**
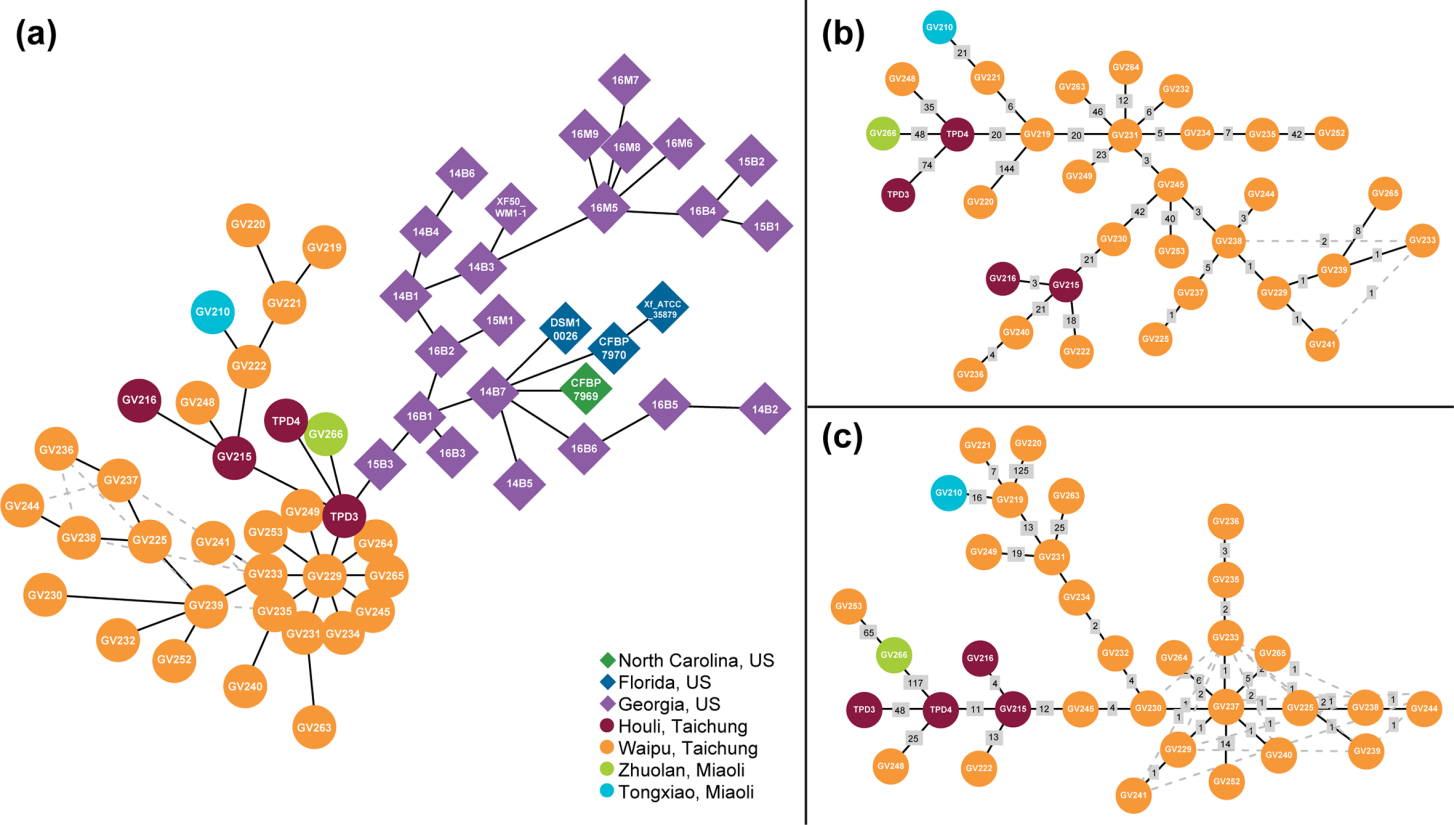
Haplotype networks showing relationships among PD-causing isolates. Isolate ID is included inside each circle/diamond. (a) Haplotype network between Taiwan (descendant population) and PD-I/Southeast USA (ancestral population) isolates. Network was built based on the core-genome alignment following removal of recombinant segments of the PD-I/Taiwan data set. (b) Haplotype network of Taiwan isolates. Network was built based on the core-genome alignment of the Taiwan-only data set. (c) Non-recombinant haplotype network of Taiwan isolates. Network was built based on the core-genome alignment following removal of recombinant segments of the Taiwan-only data set. Numbers of mutations between isolates are shown in grey boxes. The minimum spanning tree is illustrated with solid black lines. Alternative relationships among isolates are illustrated with dashed grey lines.

The core genome phylogenetic analyses of 206 worldwide isolates (Figs 1 and S2) provided strong support to the hypothesis that the introduction of subsp. *fastidiosa* into Taiwan originated from the PD-I lineage in the USA [44, 45]. All 33 Taiwanese isolates, including those two used in the previous studies to propose this hypothesis and an additional 31 newly sequenced in this study, form a monophyletic clade with the PD-I lineage. This clade is robust with 100 % bootstrap support when the entire core genome was used (Fig. 1) or when the possible recombinant segments were excluded (Fig. S2), suggesting a single source of introduction from the Southeast USA into Taiwan. Consistent with the phylogenetic relationships, nearly all isolates within this PD-I/Taiwan clade were classified as ST2 based on an established MLST scheme [66, 67]. Isolate GV225 is the only exception that was unclassified (Table S1). For closer examinations within this clade, the branch lengths are mostly very short, which demonstrates that there is little sequence differentiation. This finding suggests that the introduction event into Taiwan occurred recently, which is consistent with local PD monitoring [43, 48]. Comparatively longer branch lengths among GV219, GV229, GV230, and GV241 were observed in the phylogenetic tree. These four isolates form a monophyletic clade with isolate 14B4 from Georgia (Fig. 1a). Nonetheless, this clade is not observed when recombinant segments are removed from the core-genome alignment of all worldwide PD-causing isolates (Fig. S2). When the PD-I/Taiwan set was independently analysed using a haplotype network, two clearly differentiated groups between the Southeast USA (PD-I) and Taiwan populations were observed (Fig. 2a).

A population structure analysis using a non-recombinant core SNP alignment of all worldwide PD-causing isolates (Fig. S3) supports the conclusion that isolates from the PD-I clade and Taiwan are closely related. However, isolates GV215, GV216, GV230, GV235, and GV240 formed a distinct population (Fig. S3, shown in lime green) related to isolate CFBP7969 from North Carolina (USA). In addition, isolates TPD3, TPD4, and GV249 were also assigned to other populations. TPD3 was assigned to the same population as Je57 (PD-II from California, USA), while TPD4 was assigned to the same population as Hopland (PD-III from California, USA), XYL1732 (PD-II from Mallorca, Spain), GB514 (PD-II from Texas, USA) and 16M2 (previously assigned to PD-III from Georgia, USA). No other isolate in this data set was assigned to the same population as GV249. Overall, the population structure analysis identified 18 different populations among the worldwide set of 206 isolates. Nonetheless, most isolates were assigned to five major groups: two for the PD-I clade (Fig. S3, shown in dark blue and lime green), one for the PD-II clade (Fig. S3, shown in orange) and two in the PD-III clade (Fig. S3, shown in red and yellow). Other identified populations were usually composed of three or fewer isolates and could largely be explained by higher sequence divergence.

### The PD-causing population in Taiwan is diversifying

The core genome (i.e. shared by >99 % of isolates) of the Taiwan-only set included 2041 genes, while the soft-core genome (95–99 % of isolates) included 67 genes (Table 2). No genes were found to be uniquely present or absent in all Taiwanese isolates in respect to the Southeast USA (i.e. PD-I). In other words, there is no gene present in all (or most) Taiwanese isolates that is absent in all (or most) PD-I isolates. Similarly, there is no gene absent in all (or most) Taiwanese isolates that is present in all (or most) PD-I isolates. This finding suggests that there is no clear change in gene content linked to the introduction event. The core-genome haplotype network for the Taiwan-only population (Fig. 2b) showed that several mutations have accumulated within the region without a clear geographic pattern. The non-recombinant core-genome haplotype network confirms this trend (Fig. 2c), but it also suggests that many of these mutations are likely a product of recombinant events.

**Table 2.** Summary of pan-genome analysis of worldwide PD-causing *X. fastidiosa* data and the Taiwanese population alone. Threshold for inclusion indicates the proportion of isolates harbouring the genes

Threshold for inclusion	Worldwide (*N*=206)	Taiwan-only (*N*=33)
Core (>99 %)	1715	2041
Soft-core (95–99 %)	258	67
Shell (15–95 %)	652	272
Cloud (<15 %)	7379	757

Overall, the genetic diversity between Southeast USA (PD-I) (336 SNPs, π=2.645×10e^−05^) and Taiwan (362 SNPs, π=3.045×10e^−05^) was comparable. Likewise, the mutation rate was similar between these geographic areas (Southeast USA’s *θ*=4.986×10e^−05^ and Taiwan’s *θ*=5.101×10e^−05^). The similarities remained following removal of recombinant segments from the PD-I/Taiwan set (Table 3). The negative Tajima’s D values detected are indicative of a recent population introduction; however, values were less negative in Taiwan (−1.547) compared to the Southeast USA (−1.857). This trend was flipped when recombinant segments were removed (−2.368 for Southeast USA and −2.717 for Taiwan).

**Table 3.** Genome-wide genetic diversity measurements

Analysis	Population (*N*)	Alignment length (bp)	SNPs	Pi	Watterson	Tajima
With recombination						
PD-causing isolates+Costa Rica	Southeast USA (32)	1 562 792	926	1.346×10e^−04^	1.471×10e^−04^	−0.329
	Taiwan (33)		215	1.056×10e^−05^	3.390×10e^−05^	−2.629
PD-I/Taiwan	Southeast USA (28)	1 748 449	336	2.645×10e^−05^	4.986×10e^−05^	−1.857
	Taiwan (33)		362	3.045×10e^−05^	5.101×10e^−05^	−1.547
Without recombination						
PD-causing isolates+Costa Rica	Southeast USA (32)	1 410 222	864	1.331×10e^−04^	1.521×10e^−04^	−0.485
	Taiwan (33)		193	1.044×10e^−05^	3.372×10e^−05^	−2.633
PD-I/Taiwan	Southeast USA (28)	1 624 189	272	1.739×10e^−05^	4.345×10e^−05^	−2.368
	Taiwan (33)		398	1.767×10e^−05^	6.038×10e^−05^	−2.717

**Fig. 3.**
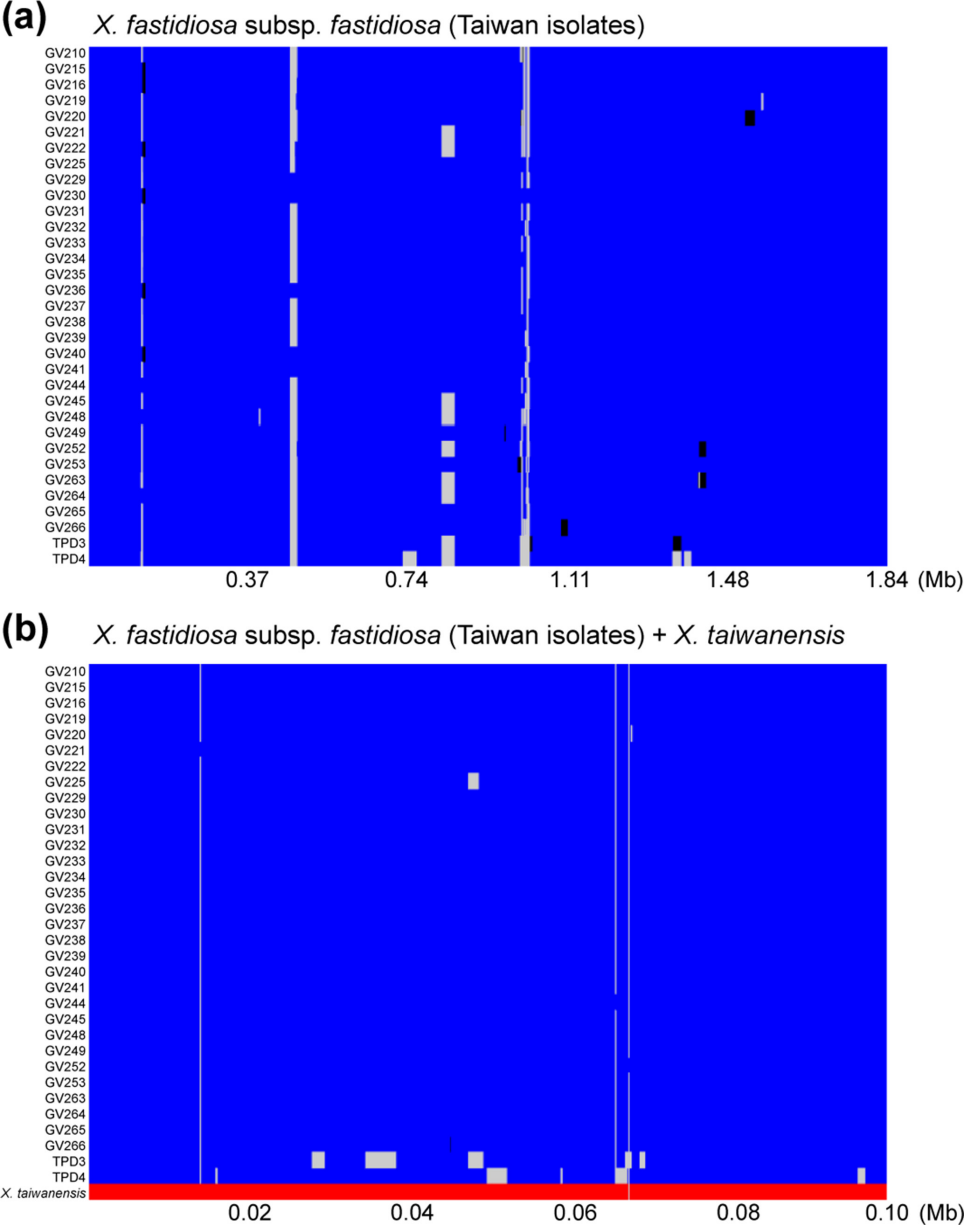
Recombination events and lineage assignments based on the core genome. Recombination events are shown across the length of the core-genome alignments. Larger areas in blue (i.e. a single lineage for all subsp. *fastidiosa* isolates in Taiwan) or red (i.e. a separate lineage for *X. taiwanensis *) represent recipient sequences. Recombinant segments from unidentified lineages are shown in black and alignment gaps are shown in grey. (a) All 33 subsp. *fastidiosa* isolates in Taiwan. The core-genome alignment contains 1 842 804 aligned nucleotide sites. (b) All 33 subsp. *fastidiosa* isolates in Taiwan and *X. taiwanensis *. The core-genome alignment contains 98 310 aligned nucleotide sites.

Based on the fastGEAR inference of core-genome recombination among Taiwanese isolates, all isolates belonged to one single lineage and there was no clear geographic structuring (Fig. 3a). This observation may or may not be attributed to the sampling bias that 82 %(27/33) of these isolates were collected from the same district. Some recent recombination events involving unknown donors (illustrated by the black blocks in Fig. 3a) were inferred. Our blastn analysis found that these recombinant segments have 100 % sequence identity with other subsp. *fastidiosa* isolates and ~98 % identity with isolates belonging to subsp. *pauca* or *multiplex*. This result suggests that not all sequence diversity found within the region has been sampled. When the sympatric sister species *X. taiwanensis* was included in the inference, the size of core genome is greatly reduced (i.e. from 1.84 to 0.1 Mb) and no inter-species recombination was identified (Fig. 3b).

## Discussion

### PD-causing subsp. *fastidiosa* was introduced into Taiwan from the Southeast USA

Despite attempts at disease surveillance, quarantine and control, novel plant pathogen outbreaks are still observed in numerous locations worldwide [23, 79–82]. Following introduction, the reconstruction of invasion routes, the identification of a pathogen’s establishment and spread mechanisms becomes crucial for developing mitigation strategies [83]. Here, we have confirmed previous studies suggesting that PD-causing subsp. *fastidiosa* was introduced to Taiwan from the Southeast USA (PD-I) [44, 45] using multiple lines of evidence based on whole-genome sequence data. The results support that the ancestor/descendant relationship between these populations was not an artefact caused by limited sampling of only two isolates from Houli in the previous study [45].

All except one of the PD-I and Taiwan isolates included here were classified as ST2 based on an established MLST scheme [66, 67] (Table S1). In addition, whole-genome-based phylogenetic analyses show that the Taiwan and Southeast USA (PD-I) isolates form one strongly supported monophyletic clade. Moreover, short branch length and lack of phylogenetically supported geographic clusters are indicative of few nucleotide changes differentially accumulating in both populations. This finding is consistent with reports of other introduction events involving *X. fastidiosa* [30, 37, 38, 42, 84–88]. Likewise, nucleotide diversity and mutation rate were comparable between Southeast USA (PD-I) and Taiwan isolates. Previous studies have found that introduced populations of subsp. *pauca* and subsp. *fastidiosa* have lower genetic diversity than native ones [34]. Yet, with time, nucleotide differences accumulate as a product of ecological and environmentally mediated pressures as well as the result of non-adaptive evolution [45]. This has been observed in the introduction of subsp. *fastidiosa* into the USA ~150 years ago [36]. Therefore, the identification of the same MLST sequence type in both Taiwan and the Southeastern USA (PD-I), the lack of genome-wide nucleotide differences among the two populations, and the similar genetic diversity and mutation rate between Taiwan and the Southeast USA (PD-I) are all indicative that this was a recent introduction event.

A previous study conducted using only two isolates from Taiwan found four putative gene gains and five putative losses in respect to the ancestral Southeast USA (PD-I) population [45]. Our updated pangenome analysis used 33 isolates from Taiwan but failed to find a gene gain/loss pattern that could be linked to this introduction. This suggests that the previous observation was an artefact of the small sample size and that no clear gene gain/loss trends can be attributed to a founder effect. Furthermore, since gene gain/loss often precedes nucleotide substitutions and indels in bacterial genome evolution [89], this is consistent with the record of invasion of *X. fastidiosa* to Taiwan in 2002. However, one caveat is that most of the genome sequences included in this gene content analysis are incomplete draft assemblies, such that the finding should be interpreted with caution.

Although a single source population (i.e. PD-I in Southeast USA) was inferred, it is difficult to determine if a single or multiple introduction events into Taiwan have taken place. Phylogenetic analyses of all worldwide PD-causing isolates and the haplotype network analysis conducted on the PD-I/Taiwan clade are indicative of a single introduction event. On the other hand, population structure analyses show that two isolates from Houli (GV215 and GV216), and three isolates from Waipu (GV230, GV235 and GV240) form a distinct population with CFBP7969 from North Carolina (USA), hinting to multiple introduction events. These isolates are neither phylogenetically nor geographically aggregated, so it is difficult to confidently ascertain if this finding is due to the differentiation of a genetically unique population in Taiwan, or if it is the product of an additional introduction event. In previous studies assessing the introduction of subsp. *multiplex* into Europe, both phylogenetic and genetic diversity data supported the hypothesis of multiple introductions from the USA [40]. Here, it is possible that our conflicting results are linked to genetic diversity losses associated with a founder event masking the presence of simultaneously introduced strains. Moreover, since most isolates were collected from the two neighbouring districts in Taichung City, it is possible that additional introduction events might have not been detected. Although additional samples from other regions in Taiwan can help to further investigate this question, such effort may be difficult following the successful PD control in recent years.

### Taiwanese isolates are differentiating via multiple mechanisms

It should be noted that despite evidence suggesting a short evolutionary time since the introduction of PD-causing subsp. *fastidiosa* to Taiwan, sequence divergence in core-genome genes is already observed in the region. It is not possible to confidently determine if this is due to variable evolutionary pressures across locations in Taiwan or an intrinsic genetic characteristic carried over from the ancestor population(s). Waipu and Houli are neighbouring districts that are <10 km apart and share similar warm humid subtropical climate conditions, so it would be expected that environmental pressures would be similar among populations. Likewise, Zhuolan and Tongxiao are separated from Waipu and Houli by only ~20 km. This proximity could also explain the lack of clear phylogenetic differentiation among isolates from distinct locations.

Another notable aspect is how the haplotype network analysis responded to the removal of recombinant regions. When recombinant segments were included, the minimum spanning tree (solid black lines) was defined and showed no clear separation among Taiwanese isolates from different locations. Following the removal of recombinant segments, Houli isolates clustered within the network and the relationships among Waipu isolates became less clear (dashed grey lines). This is evidence that recombination significantly contributes to the genetic differentiation of PD-causing subsp. *fastidiosa* in Taiwan; however, this result could also be sensitive to low sample size in certain regions. The removal of recombinant segments from the worldwide PD-causing core-genome alignments also had an effect in the topology of the non-recombinant phylogenetic tree of worldwide PD-causing isolates. Mainly, a monophyletic clade grouping GV219, GV229, GV230, and GV241 with 14B4 from Georgia was absent from the non-recombinant tree of worldwide PD-causing isolates. This provides further support that recombination plays a significant role in the diversification of the Taiwanese population. In addition, it also suggests that, to some degree, the recombinant capacity of some Taiwanese PD-causing isolates might be linked to the isolates introduced from the USA.

Previous studies have shown that natural competency and recombinant capacity are isolate dependent [37, 38, 40, 90]. Moreover, recombinant events can be carried over from ancestral populations into new introductions; particularly, in instances where these introductions are recent [40]. Therefore, our results are indicative that recombinant-prone subsp. *fastidiosa* isolates might have been introduced to Taiwan from the Southeast USA or that they might have evolved there. Further sampling from additional regions in Taiwan would be required to evaluate this. Nonetheless, the existence of unknown recombining sequences in Taiwanese isolates suggest that genetic diversity within Taiwan is higher than what can be defined via our current sampling. Finally, even though homologous recombination is known to occur in sympatric subsp. *pauca* populations [91] and among different *X. fastidiosa* subspecies [38], no recombination events between PD-causing subsp. *fastidiosa* and *X. taiwanensis* were detected. This result is expected based on the low level of genome-wide nucleotide sequence identity between these two species at ~84 % [52, 53] and further supports that these two species are ecologically and/or biologically isolated despite diseased grapevines and pears can be found in proximity in Central Taiwan.

## Conclusion

In globally distributed pathogens, understanding the relationships among populations and the location-specific mechanisms of diversification has significant implications in the development of successful management techniques. Here, we used phylogenetic analysis to investigate the introduction of subsp. *fastidiosa* into Taiwan and confirm that the invasion originated from a single-source population (i.e. the Southeast USA). Haplotype network analysis, pan-genome trends, and global genetic diversity measures support this hypothesis. Following its recent establishment within Taiwan, PD-causing subsp. *fastidiosa* has differentiated across geographic locations. We propose that part of this diversification can be linked to homologous recombination and highlight the need for further studies within the region. Moreover, focus should be allocated in acquiring further samples from other location within Taiwan and addressing how natural selection, and other evolutionary forces, might impact local adaptation across regions.

## Supplementary Data

Supplementary material 1

Supplementary material 2
