# Supplementary material for: Genetic differentiation of Xylella fastidiosa following the introduction into Taiwan
Source: Microb Genom. 2021 Dec 13;7(12):000727. doi: 10.1099/mgen.0.000727 (PMC8767338; doi:10.1099/mgen.0.000727)
Supplement: Supplementary material 1 [file mgen-7-0727-s001.pdf]

Supplementary Materials

Genetic differentiation of *Xylella fastidiosa* following the introduction into Taiwan

Andreina I. Castillo [1#], Chi-Wei Tsai [2#], Chiou-Chu Su [3], Ling-Wei Weng [2], Yu-Chen Lin [4], Shu-Ting Cho [4], Rodrigo P. P. Almeida [1], Chih-Horng Kuo [4*]

[1] Department of Environmental Science, Policy and Management, University of California, Berkeley, CA 94720, USA
[2] Department of Entomology, National Taiwan University, Taipei 106, Taiwan
[3] Division of Pesticide Application, Taiwan Agricultural Chemicals and Toxic Substances Research Institute, Taichung 413, Taiwan
[4] Institute of Plant and Microbial Biology, Academia Sinica, Taipei 115, Taiwan

[#] Equal contribution
* Corresponding author: Chih-Horng Kuo; chk@gate.sinica.edu.tw

Table S1. Metadata of all *Xylella* isolates included in this study.
(Available as a separate Excel file)

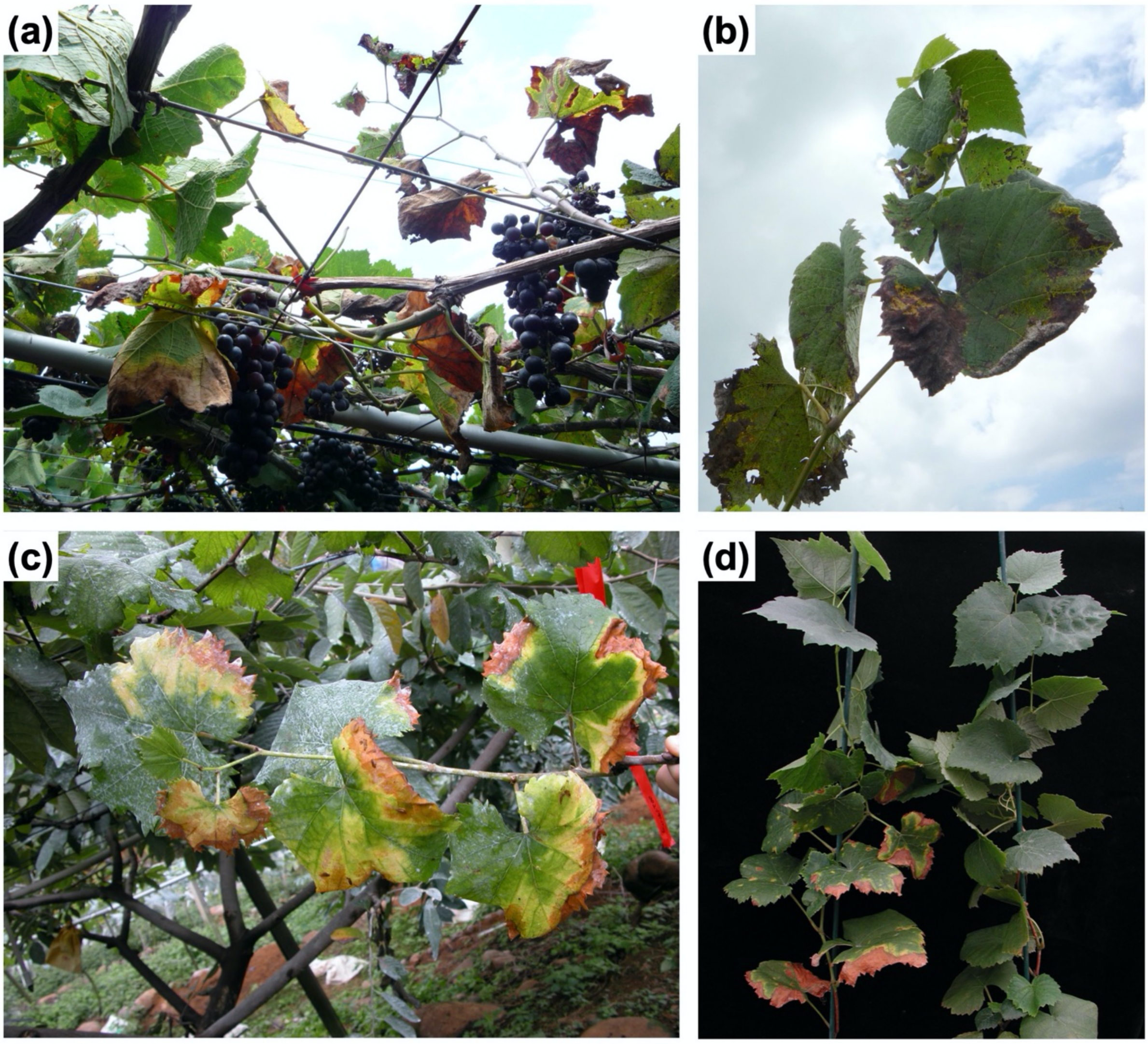

Figure S1. Symptoms of Pierce's disease on grapevine cultivars. (a) Black Queen. **(b)** Golden Muscat. **(c)** Kyoho. **(d)** Golden Muscat inoculated with *Xylella fastidiosa* subsp. *fastidiosa* (left) and mock (right).

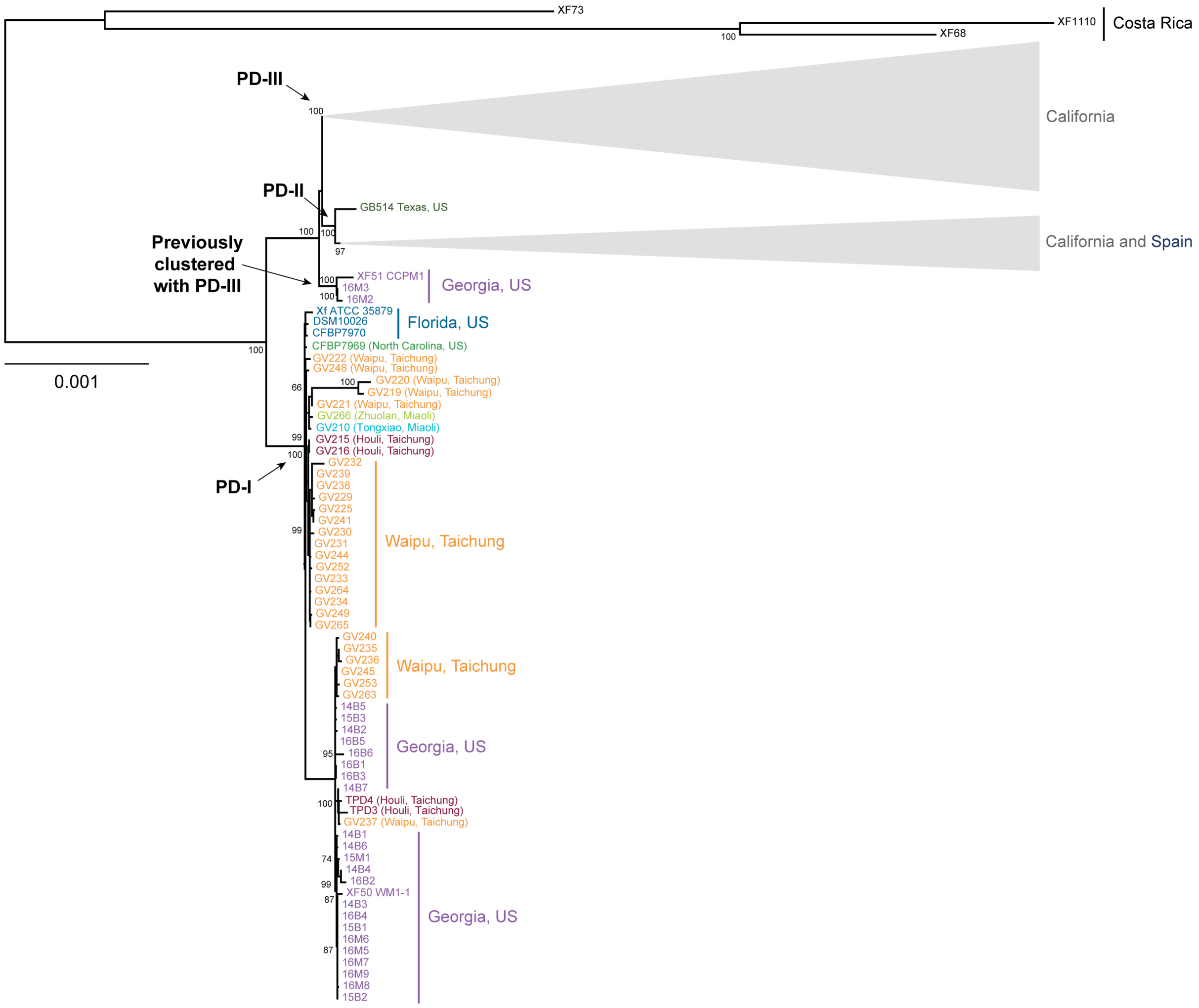

Figure S2. Maximum Likelihood non-recombinant tree of worldwide PD-causing subsp. *fastidiosa* isolates. The phylogeny was constructed using the core genome excluding the recombinant segments detected by fastGEAR. Costa Rican isolates are used to root the tree. Clades encompassing California and Spain isolates have been compressed to their most recent common ancestor. The points of divergence for the PD-I to PD-III clades are indicated by arrows. Distinct colors are used to differentiate isolates from different geographic regions.

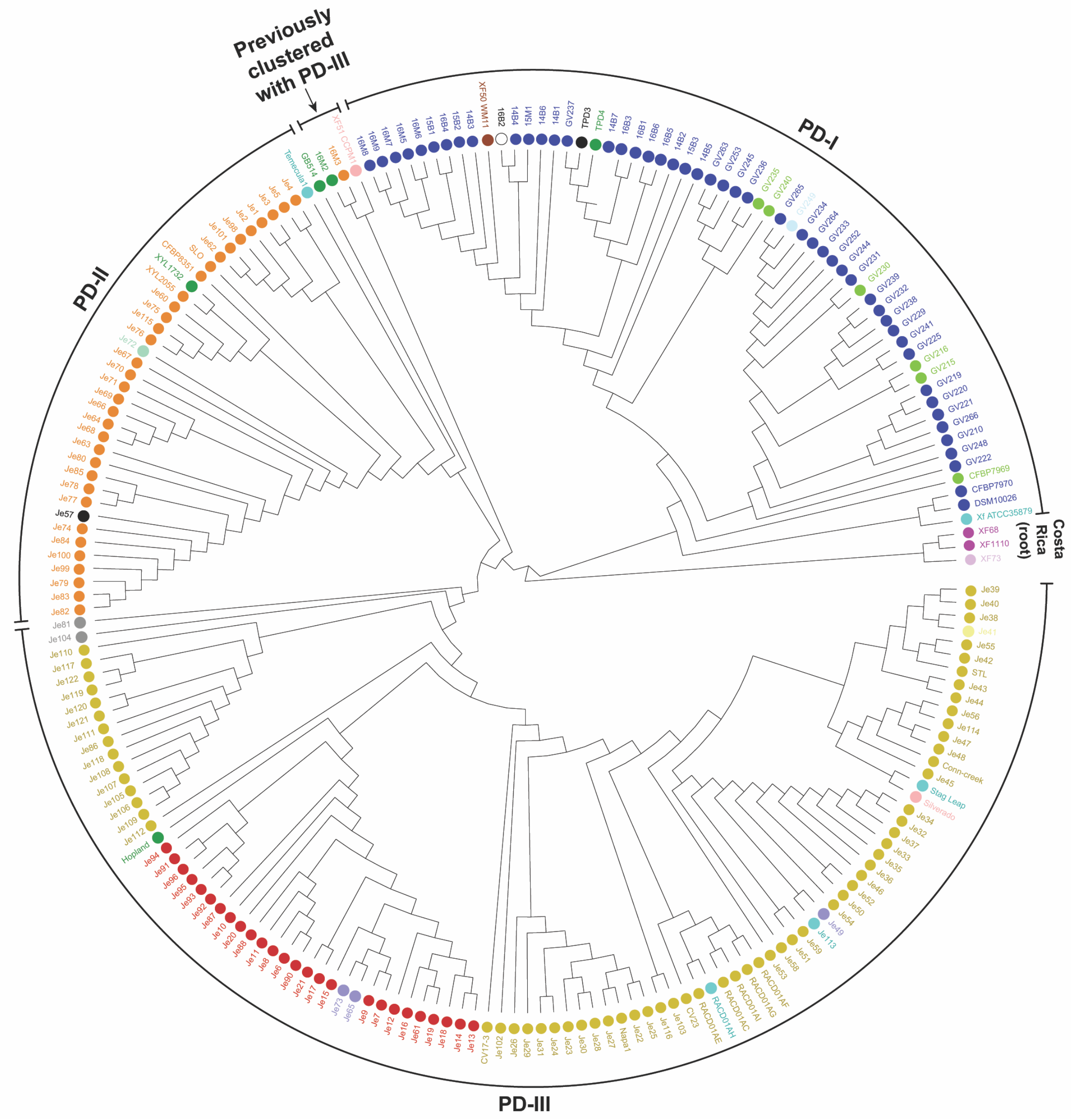

Figure S3. Evolutionary relationships among of populations. The maximum likelihood cladogram was generated by hierBAPS based on a non-recombinant SNP alignment of the core genome. A total of 18 populations, as defined by hierBAPS at level 2, are distinguished by distinct colors for the isolate names shown at the tip; more detailed information about the five major populations (shown in dark blue, lime green, orange, red, and yellow) is described in the main text. The three phylogenetic clades defined in previous studies are labelled in the out-most ring.
